# Efficacy of brow suspension with autogenous fascia lata in simple congenital ptosis

**DOI:** 10.12669/pjms.332.11521

**Published:** 2017

**Authors:** Farhan Ali, Muhammad Saim Khan, Muhammad Sharjeel, Zaheer Ud Din, Bilal Murtaza, Asfandyar Khan

**Affiliations:** 1 Dr. Farhan Ali, MBBS, FCPS. Mayo Hospital, King Edwards Medical University, Lahore, Pakistan; 2 Dr. Muhammad Saim Khan, MBBS, FCPS, FICO, MRCSEd. Armed Forces Institute of Ophthalmology, Rawalpindi, Pakistan; 3 Dr. Muhammad Sharjeel, MBBS, FCPS. Mayo Hospital, King Edwards Medical University, Lahore, Pakistan; 4 Dr. Zaheer Ud Din, MBBS, FCPS. Armed Forces Institute of Ophthalmology, Rawalpindi, Pakistan; 5 Dr. Bilal Murtaza, MBBS. Armed Forces Institute of Ophthalmology, Rawalpindi, Pakistan; 6 Dr. Asfandyar Khan, MBBS, FCPS. Armed Forces Institute of Ophthalmology, Rawalpindi, Pakistan

**Keywords:** Fascia Lata Sling, Interpalpebral Fissure Height, Ptosis

## Abstract

**Objective:**

To assess the mean change in interpalpebral fissure height and marginal reflex distance after brow suspension with autogenous fascia lata sling in patients of ptosis.

**Methods:**

This was a Quasi experimental study conducted at Department of Ophthalmology, Mayo Hospital, King Edwards Medical University Lahore, from Jan 2013 to June 2016. Included were the patients who had unilateral or bilateral ptosis with poor levator function (< 5 mm). Informed consent was obtained from all patients after explaining about the research project. Patients were admitted in ward and all of them underwent surgery by a single surgical team. The surgical procedure was performed in supine position under general anesthesia in children and uncooperative patients. Patients were followed at week 4, 8, 12 and 24 to observe vertical interpalpebral fissure height and marginal reflex distance.

**Results:**

The mean age of the patients was 9.03 ± 5.26 years. The mean Inter palpebral fissure height (IPFH) was 4.40±0.91 mm and mean MRD was 0.50 ± 1.00 mm before surgery while after surgery it was 7.41±0.76 mm and 3.10 ± 1.50 mm respectively at 04 weeks. The mean IPFH and MRD at 24 weeks postoperatively were 8.43±0.98 mm and 3.60 + 1.50 mm respectively. The mean change in IPFH and MRD at 24^th^ week, were 3.90 ± 0.34 mm and 3.50 ± 1.00 mm.

**Conclusion:**

Brow suspension with fascia lata sling is safe and effective technique for correction of ptosis with poor levator function.

## INTRODUCTION

Ptosis, also named as blepharoptosis, is an ocular condition in which the upper lid margin is abnormally lower than its normal location with the eye in primary gaze. The normal adult upper lid is highest nasal to the pupil and covers 1-1.5 mm below the superior limbus.^1^,^2^ The basic etiology of ptosis is weakness of either of two elevators of the upper lid that include levator palpebrae superioris and muller muscle. Ptosis can be categorized into congenital and acquired subtypes. It can also be classified on the basis of etiology into various groups such as myogenic, mechanical, neurogenic, aponeurotic and traumatic.^3^-^6^

Management of ptosis include medical treatment or surgery.^7^,^8^ Out of surgical procedures, Various surgical techniques can be used to correct ptosis depending upon the severity of ptosis and levator function. These various surgical procedures such as Muller muscle resection, Fasnella Servat procedure, levator aponeurosis advancement, levator resection and brow suspension are performed considering into account primarily the levator function.^9^,^10^

Frontalis suspension also known as brow suspension is a commonly performed surgical procedure for correction of severe ptosis with poor levator function. In this surgery sling material is used to connect the eyelid to the brow and power of the frontalis muscle is utilized to elevate the poorly functioning eyelid. Various materials have been tried for brow suspension such as muscle tendon, preserved donor sclera, artificial materials that included catgut or silk sutures, wires of gold, silver or platinum metals, however the most widely used artificial material is polyfilament suture which is easy to handle with lesser incidence of complications.^11^-^14^ Brow suspension with autogenous fascia lata is considered as gold standard procedure for congenital ptosis as it has a long-lasting effect for upper eyelid elevation. When a piece of free autologous fascia is transplanted to the eyelid, it can easily survive. Various comparative studies have shown that using a fascia lata sling is more effective than other materials and the incidence of complications such as infections, extrusion, breakage and granuloma formation is lower than other materials.^15^-^17^

Rationale of conducting this study was to assess the efficacy of brow suspension with fascia lata by measuring the mean change inter-palpebral fissure height in simple congenital ptosis. Multiple studies have been conducted in various part of the world to assess the efficacy of brow suspension but to the best of our knowledge no study has been conducted in our part of the world. This will be 1^st^ study in our population to assess the use of fascia lata sling for correction of ptosis. This will also help to improve patients’ satisfaction as well as our knowledge and practice.

## METHODS

This was a Quasi experimental study conducted at Department of Ophthalmology, Mayo Hospital, King Edwards Medical University, Lahore from January 2013 to June 2016. Patients with age ranging from 5 - 26 years and diagnosed as having unilateral or bilateral ptosis with poor levator function were included by non probability purposive sampling. All those patients who had other associated conditions such as poor Bells’ phenomenon, lagophthalmos, Horner syndrome, Chronic progressive external ophthalmoplegia, Myasthenia gravis, Marcus gunn jaw winking syndrome, Blephrophimosis syndrome, third nerve palsy or traumatic ptosis were excluded from the study. Open EPI calculator was used to calculate the sample size which appeared to be at least 60 eyes. After taking permission from hospital ethical committee, 92 eyes of 70 patients initially fulfilled the selection criteria and registered in the study but 08 patients fail to have regular follow up and finally enrolled in the study were 80 eyes of 62 patients. Informed consent was obtained from all patients after explaining about the research project. Demographic details were documented, detailed ocular and systemic examination was carried out and measurements of ptosis were recorded on patients` proforma. Preoperative general anesthesia fitness was checked by anesthesiologist. All the patients underwent surgery by a single surgical team. Patients were followed at week 4, 8, 12 and 24 weeks, IPFH and marginal reflex distance (MRD) was documented.

### Procedure

Patients were admitted to indoor facility. one day before the day of surgery. The surgical procedure was performed in supine position under general anesthesia by single surgical team. Fox pentagon technique was used for brow suspension and autologous fascia lata was used which was harvested from patients thigh. Two stab incisions were made through skin and orbicularis to reach tarsal plate. Another two incisions were made in the upper hair line of brow aligned with medial and lateral canthus and finally a third incision was made a finger breadth (8-10mm) above the two brow incision. Fascia lata after being harvested from thigh was passed in the sub mascular plane and secured by a knot in the superior incision. After the surgery patients were advised oral antibiotics and analgesics. Topical antibiotics and lubricants were used as a routine in all the patients for two weeks.

### Statistical Analysis

Statistical package for social sciences (SPSS) 22.0 was used for statistical analysis of the data. Quantitative variables like age, vertical interpalpebral fissure height (IPFH) and marginal reflex distance (MRD) were analyzed before and after surgery at 4, 8, 12 and 24 weeks and presented in form of mean ± S.D. Qualitative variables like gender and laterality were presented in form of frequency and percentage. Mean pre-operative and post-operative vertical IPFH and MRD were compared by using paired sample t-test taking p-value <0.05 as significant. Mean induced change in IPFH and MRD were computed from the difference of preoperative and postoperative measurements.

## RESULTS

**Fig.1:**
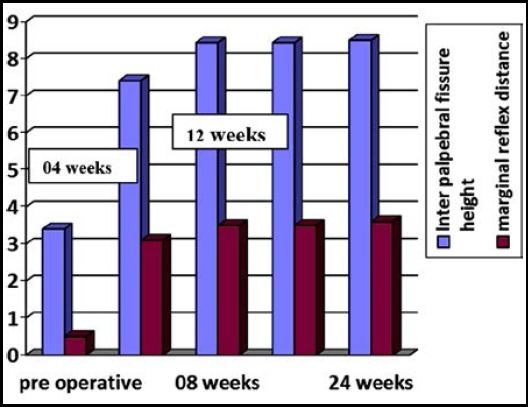
IPFH and MRD before and after surgery.

A total of 80 eyes of 62 patients (18 (29%) bilateral and 44 (71%) unilateral) underwent ptosis surgery. Out of total 67% were male while 33% were females and mean age of the patients was 9.03 ± 5.26 years.(Table-1) Preoperative mean IPFH was 4.40±0.910 mm and mean MRD was 0.50 ± 1.00 mm and there was statistically significant increase in IPFH and MRD after surgery at 04 weeks as shown in Table-II. The pre and postoperative IPFH and MRD are depicted in Fig.1. The induced change in IPFH at 24 weeks postoperatively ranged from 3-8 mm with a mean of 3.90 ± 0.34 mm while in MRD, it varied between 1-4 mm with a mean of 3.50 ± 1.00 mm.

**Table-I:** Frequency distribution of Gender and Laterality.

*Variables*		*Frequency*	*Percentage*
Gender	Male	41	67 %
	Female	21	33 %
Laterality	Right	20	34%
	Left	24	38%
	Both eyes	18	29%

**Table-II:** Mean and standard deviation of continuous variables.

*Variable*	*N*	*Mean ± SD*	*P- value*
Age	80	9.03 ± 5.26 years	-
Preoperative IPFH	80	4.40±0.910	-
Preoperative MRD	80	0.50 ± 1.00	-
Postoperative IPFH (04 weeks)	80	7.41±0.76 mm	P < 0.001
Postoperative MRD (04 weeks)	80	3.10 ± 1.50 mm	P < 0.001

## DISCUSSION

Congenital ptosis is the most common type of ptosis with no predilection for any gender or any of the two eyes.^17^ We, in this study could not find out any statistically significant difference in the incidence or severity of ptosis between males and females. The involvement of either eye (right or left eye) was also statistically insignificant. The gold standard surgical procedure for severe ptosis with poor levator function is brow suspension with autogenous facia lata.^18^ Frontalis suspension with autogenous facia lata is most popular procedure owing to its safety, efficacy and least incidence of complications. However, in patients less than three years old, this surgical technique cannot be possible because autogenous fascia lata is not developed adequately by this age.^18^-^20^

Like Kasturi Bhattacharjee et al, in this study, improvement in IPFH and MRD was statistically as well as clinically significant.^21^ Ninety two percent (58 patients) of our patients were satisfied with the cosmetic and functional outcome of the surgery. Compensatory head posture (chin elevation) was relieved in almost all the patients. The final height of the eyelid was achieved at 08 weeks and that remained same at 12 and 24 weeks. Like other studies we intended to elevate the lid upto the superior limbus in all the patients.^22^ Postoperative lid lag which was expected to occur as a result of lack of complete release of lid was found only in 08 patients. However, none of the patients develop any signs of exposure because patients with poor Bells’ phenomenon were excluded in the first place.

Despite its safety profile, studies have shown that brow suspension with facia lata may be associated with complications especially when a larger area is removed. The documented complications that can occur include hematoma formation, infection, sepsis and scar formation at the harvested site.^22^,^23^ Few of our patients complained of transient pain at the harvested facia lata site and walking but that resolved with postoperative oral antibitics and analgesics within a week. Four (8%) of our patients had poor surgical and cosmetic outcome and a redo surgery was performed, however none of the patient developed forehead granuloma, cosmetically disfiguring scar, exposure of the knot or fistula formation.

Currently available literature on the use of autogenous facia lata in ptosis surgery made us carry out this study in our population. Considering the results of our study we recommend this procedure to be the ‘procedure of choice’ in severe congenital ptosis with poor levator function. It is worth mentioning that other materials (both natural or artificial) that can be used in place of facia lata are also commonly practiced all over the world especially when the surgery is required in relatively younger age group of 03 years or less. Therefore, further studies comparing the use of facia lata with its alternatives may need to be carried out in our population on a larger cohort and for longer duration, so that to infer more comprehensive results.

The results of our study and the outcome of brow suspension with facia lata in our population are comparable to internationally published literature. We conclude that brow suspension with facia lata is a safe and effective procedure with desired anatomical and cosmetic outcome. We also found out that patient selection is very important and therefore, a detailed preoperative assessment of ptosis especially to rule out associated superior rectus weakness, poor Bells` phenomenon and other associated systemic and ocular disorders must be carried out in every patient. This practice will produce better results both anatomically as well as cosmetically and reduce the chances of associated complications.

### Authors’ Contribution

***Farhan Ali:*** Conception, Data collection.

***M Saim Khan:*** Data analysis and drafting the manuscript.

***M Sharjeel:*** Design & Data collection.

***Bilal Murtaza:*** Data collection.

***Asfandyar Khan:*** Data collection and analysis.

***Zaheer-ud-Din:*** Final review.

## References

[1] Zoumalan CI, Lisman RD (2010). Evaluation and management of unilateral ptosis and avoiding contralateral ptosis. Aesthetic Surg J.

[2] De Sanctis U, Alovisi C, Actis AG, Vinai L, Penna R, Fea A (2013). Blepharoptosis. Minerva Chirurgica.

[3] Finsterer J (2003). Ptosis:causes, presentation, and management. Aesthetic Plastic Surg.

[4] Gomez J, Laquis SJ (2015). Blepharoptosis:Clinical Presentation, Diagnosis, and Treatment. Insight.

[5] Lim JM, Hou JH, Singa RM, Aakalu VK, Setabutr P (2013). Relative incidence of blepharoptosis subtypes in an oculoplastics practice at a tertiary care center. Orbit.

[6] Frueh BR (1980). The mechanistic classification of ptosis. Ophthalmology.

[7] Sakol PJ, Mannor G, Massaro BM (1999). Congenital and acquired blepharoptosis. Curr Opin Ophthalmol.

[8] Malik KJ, Lee MS, Park DJ, Harrison AR (2007). Lash ptosis in congenital and acquired blepharoptosis. Arch Ophthalmol.

[9] Emsen IM (2008). A new ptosis correction technique:a modification of levator aponeurosis advancement. J Craniofacial Surg.

[10] Waqar S, McMurray C, Madge SN (2010). Transcutaneous blepharoptosis surgery - advancement of levator aponeurosis. Open Ophthalmol J.

[11] Salvi SM, Currie ZI (2009). Frontalis suspension sling using palmaris longus tendon in chronic progressive external ophthalmoplegia. Ophthalmic Plastic Reconst Surg.

[12] Bodian M (1968). Repair of ptosis using human sclera. Am J Ophthalmol.

[13] Jenkins DH, Forster IW, McKibbin B, Ralis ZA (1977). Induction of tendon and ligament formation by carbon implants. J Bone Joint Surg Br vol.

[14] Katowitz JA (1979). Frontalis suspension in congenital ptosis using a polyfilament, cable-type suture. Arch Ophthalmol.

[15] Takahashi Y, Leibovitch I, Kakizaki H (2010). Frontalis suspension surgery in upper eyelid blepharoptosis. Open Ophthalmol J.

[16] Debski T, Jethon J, Pietruski P, Radzikowska E (2012). Frontalis suspension using autogenous fascia lata--evaluation of long-term outcome. Klinika Oczna.

[17] Lim JM, Hou JH, Singa RM, Aakalu VK, Setabutr P (2013). Relative incidence of blepharoptosis subtypes in an oculoplastics practice at a tertiary care center. Orbit.

[18] Bansal RK, Sharma S (2015). Results and complications of silicone frontalis sling surgery for ptosis. J Pediat Ophthalmol Strabismus.

[19] Ben Simon GJ, Macedo AA, Schwarcz RM, Wang DY, McCann JD, Goldberg RA (2005). Frontalis suspension for upper eyelid ptosis:evaluation of different surgical designs and suture material. Am J Ophthalmol.

[20] Suh JY, Ahn HB (2013). Ptosis repair using preserved fascia lata with the modified direct tarsal fixation technique. Korean J Ophthalmol.

[21] Bhattacharjee K, Bhattacharjee H, Kuri G, Shah ZT, Deori N (2013). Single-stage surgery for Blepharophimosis syndrome. Indian J Ophthalmol.

[22] Dubiel WT, Wigren A (1974). Functional status of the lower extremity after resection of fascia lata. A clinical and physiological follow-up study in patients with fascia lata heart valve replacement. Acta Orthopaedica Scandinavica.

[23] Collin JR (1979). A ptosis repair of aponeurotic defects by the posterior approach. Br J Ophthalmol.

